# Stem Cell Therapy for Microvascular Injury Associated with Ischemic Nephropathy

**DOI:** 10.3390/cells10040765

**Published:** 2021-03-31

**Authors:** Stephen C. Textor, Abdu Abumoawad, Ahmed Saad, Christopher Ferguson, Allan Dietz

**Affiliations:** 1 Mayo Clinic, Division of Nephrology and Hypertension, Rochester, MN 55905, USA; Ferguson.Christopher@mayo.edu; 2 Department of Medicine University of Missouri, Kansas, MO 64108, USA; Abumoawad.Abdelrhman@umkc.edu; 3 Department of Medicine Creighton University School of Medicine, Omaha, NE 68124, USA; AhmedSaad@creighton.edu; 4 Mayo Clinic, Human Cell Therapy Laboratory, Rochester, MN 55905, USA; Dietz.Allan@mayo.edu

**Keywords:** ischemic nephropathy, renal artery stenosis, tissue oxygenation, chronic kidney disease

## Abstract

Ischemic nephropathy reflects progressive loss of kidney function due to large vessel atherosclerotic occlusive disease. Recent studies indicate that this process is characterized by microvascular rarefaction, increased tissue hypoxia and activation of inflammatory processes of tissue injury. This review summarizes the rationale and application of functional MR imaging to evaluate tissue oxygenation in human subjects that defines the limits of renal adaptation to reduction in blood flow, development of increasingly severe tissue hypoxia and recruitment of inflammatory injury pathways in ischemic nephropathy. Human mesenchymal stromal/stem cells (MSC) are capable of modifying angiogenic pathways and immune responses, but the potency of these effects vary between individuals and various clinical characteristics including age and chronic kidney disease and levels of hypoxia. We summarize recently completed first-in-human studies applying intrarenal infusion of autologous adipose-derived MSC in human subjects with ischemic nephropathy that demonstrate a rise in blood flow and reduction in tissue hypoxia consistent with partial repair of microvascular injury, even without restoring main renal arterial blood flow. Inflammatory biomarkers in the renal vein of post-stenotic kidneys fell after MSC infusion. These changes were associated with modest but significant dose-related increments in kidney function. These data provide support a role for autologous MSC in repair of microvascular injury associated with tissue hypoxia.

## 1. Introduction

Epidemiologic studies identify loss of kidney function as a major risk factor for cardiovascular events and death. Treatment options for many forms of acute and chronic kidney disease (CKD) are limited beyond measures to reduce systemic blood pressures and avoid hyperfiltration injury. Recent studies in rodent models indicate a remarkable potential for mesenchymal stromal/stem cell-based therapies to favorably influence kidney allograft survival and recovery from acute kidney injury (AKI) and some forms of chronic kidney disease including diabetes and lupus nephritis, as has been reviewed [1]. The unique features of kidney microcirculation render the kidney particularly susceptible to ischemic injury. How these data translate to human disorders remains poorly understood. The aim of this review was to summarize the initial studies utilizing autologous MSC in human subjects with ischemic nephropathy due to atherosclerotic vascular disease of the kidney.

## 2. Renovascular Disease and Microvascular Injury

**Figure 1:**
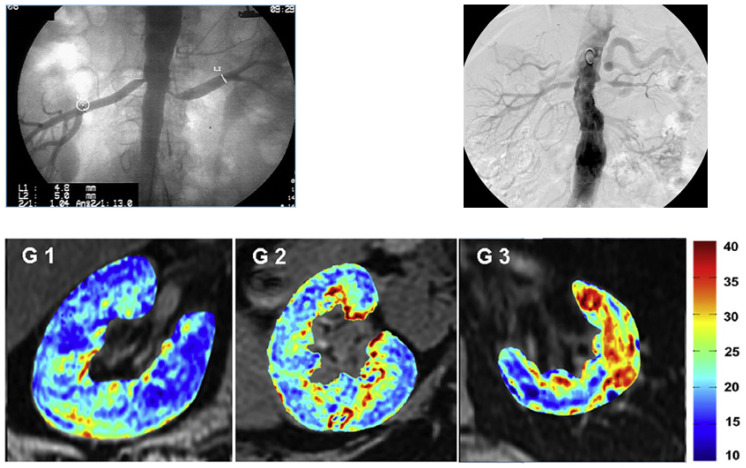
Large vessel atherosclerotic renovascular disease (ARVD) produces reduction in blood flow and loss of kidney volume and glomerular filtration rate (GFR). These changes eventually induce widening tissue hypoxia. The upper panel depicts angiograms with moderate stenoses (**left**) and more severe occlusion (**right**) associated with reduced kidney volume and declining glomerular filtration rate (GFR). The bottom panel images depict the increasing intensity of tissue deoxygenation as measured by blood-oxygen-level-dependent (BOLD) MRI in post-stenotic kidneys associated with decreasing GFR. G1: the highest quartile of 48 subjects, GFR: 51 ± 12; G2: middle two quartiles, GFR: 25 ± 6 mL/min; G3: the lowest quartile, GFR: 8 ± 3 mL/min. Data from [4] with permission. Such changes characterize ischemic nephropathy and are associated with progressive inflammation and tissue damage (see text).

Large vessel atherosclerotic renovascular disease (ARVD) is highly prevalent in older populations as a result of renal artery stenosis. This process develops gradually over many years and is accelerated by dyslipidemia, diabetes, hypertension, smoking and other atherosclerotic risk factors. When lumen occlusion reaches a “critical” threshold, usually identified as more than 70–80% vessel occlusion, distal perfusion pressures and blood flows begin to fall measurably [2] (Figure 1). Rich literature on hypertension has linked loss of renal perfusion pressure with activation of multiple pressor pathways that produce and accelerate renovascular hypertension, including the renin–angiotensin aldosterone system [3]. While main renal artery revascularization previously was the primary means of treating ARVD and renovascular hypertension, advances in antihypertensive drug therapy in recent years have made optimized medical therapy the mainstay of management and can often achieve target blood pressures successfully. One consequence of successful medical therapy has been a marked reduction in revascularization procedures. As a result, many individuals now are subjected to reduced renal blood flow and distal perfusion pressures for prolonged periods of times, sometimes many years. Sustained loss of blood flow and subsequent loss of kidney function have been designated “ischemic nephropathy.”

**Figure 2:**
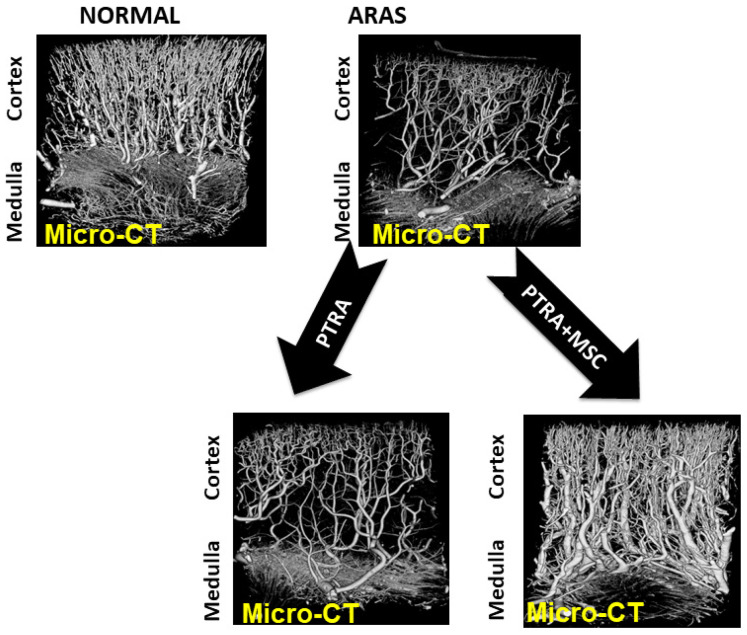
Microvascular CT imaging of the kidney in a swine model of gradually developing ARVD treated with percutaneous transluminal renal angioplasty (PTRA). These data demonstrate loss of cortical microvasculature developing in a post-stenotic kidney associated with loss of glomerular filtration rate (GFR). Importantly, restoring main renal artery patency with PTRA fails to restore the microvasculature, a similar finding to Table 67. These experimental data provided the basis for human MSC administration in ARVD. ARAS = atherosclerotic renal artery stenosis

Importantly, large vessel ARVD leads to distal microvascular disturbances with loss of medium and small diameter vessels and ultimately with parenchymal fibrosis [5,6] (Figure 2). Loss of renal microvasculature integrity has been identified as a common final pathway for other kidney diseases as well, including diabetic nephropathy, obesity and nephrosclerosis [7]. While ARVD initially manifests as a hemodynamic disorder that sometimes can be reversed with restoration of vessel patency, subsequent microvascular remodeling and rarefaction eventually fail to be reversible. A corollary observation is that even endovascular repair with arterial dilation and stenting often fails to restore full levels of blood flow and recover glomerular filtration [8,9]. Failure to recover kidney function after revascularization is associated with reduced long-term survival and increased need for renal replacement therapy such as dialysis and/or transplantation [10]. Over the past several decades, ARVD has been associated more commonly with end-stage renal disease (ESRD) and marked increases in associated cardiovascular disease events and mortality in the United States [11,12].

## 3. Ischemic Nephropathy and Renal Oxygenation

Unlike heart or brain, the whole kidney is substantially overperfused relative to its metabolic energy requirements [13]. As a result, multiple intrarenal regulatory mechanisms both limit oxygenation within the cortical segments (e.g., as a result of arteriovenous shunts and local restrictions for oxygen diffusion) and result in functional local gradients. Arteriovenous shunting reduces oxygen tensions within the renal cortex toward levels of 55–60 mm Hg, a distinct stepdown from 85–90 mm Hg in the normal renal artery [14,15]. The normally functioning kidney exhibits markedly different regional levels of tissue oxygenation due to limited post-glomerular blood flow to the medulla and enhanced oxygen consumption associated with energy-dependent tubular solute transport [15,16]. Medullary oxygenation can be as low as 10–15 mm Hg in a normally functioning state, which some argue renders it uniquely susceptible to acute kidney injury when nephrotoxic insults are superimposed [17]. Recognition of these perfusion characteristics and that ARVD is highly prevalent led some authors to designate loss of renal function beyond a stenotic lesion as ischemic nephropathy [18], assuming that loss of blood flow must induce tissue hypoxic injury [19].

Functional evaluation of kidney tissue oxygenation in human subjects has been possible only since the introduction of blood-oxygen-level-dependent magnetic resonance imaging (BOLD MRI) [20] (Figure 1). This method depends upon the paramagnetic property of deoxygenated hemoglobin that modifies magnetic realignment after a field disruption, whereas oxygenated hemoglobin has no active magnetic moment [21]. Hence, the measured rate of realignment (expressed as R2*) is a direct reflection of the proportion of deoxygenated hemoglobin within a specific voxel containing blood. The normal levels of oxygenation within the kidney vary over the steepest segments of the hemoglobin dissociation curve, making this a particularly suitable tool for this organ. Studies utilizing BOLD MRI emphasize the need for close standardization related to sodium intake, diuretic use and water ingestion as even modest changes in these parameters have measurable effects on local levels of tissue deoxygenation. These reflect alterations in oxygen consumption associated with solute and water transport in the medullary segments [22,23,24]. With careful attention to such factors, BOLD imaging allows identification of changes of cortical and medullary renal oxygenation associated with chronic kidney disease [25,26], vascular occlusive disease, drug therapy, acute kidney injury after contrast injection and other conditions [27,28].

Our research group has employed systematic BOLD imaging of the kidney in human subjects with ARVD and with essential hypertension, specifically to examine the level of ischemia associated with ARVD. These studies also allowed repeated measurements of both cortical and medullary blood flows for each kidney using central venous contrast CT imaging as previously described [29]

Initial studies in human subjects with essential hypertension and ARVD were undertaken under in-patient research protocol conditions with fixed daily sodium intake and standardized medication administration [30,31]. Overall kidney function was moderately reduced with an upper limit of serum creatinine below 2.5 mg/dL, in part due to asymmetric loss of kidney tissue associated with compensatory hypertrophy and hyperfiltration in the contralateral kidney. We anticipated that reduced blood flows would magnify measurable hypoxic injury, particularly within deep medullary regions known to have reduced oxygenation under normal conditions. Somewhat unexpectedly, these studies established a remarkable range of adaptation with preserved oxygen gradients within post-stenotic kidneys despite substantial reductions in blood flows both to the cortex and the medulla, lower kidney parenchymal volume and reduced solute filtration. Both cortical and medullary oxygenation measurements were well-preserved despite blood flow reductions of up to 40% [31]. In part, this reflected abundant reserves of oxygenation within cortical structures and reduced medullary oxygen consumption as a result of lower filtration and solute transport. These data provide physiologic confirmation of resilience within a normal human kidney that allows gradual hemodynamic compromise to develop with preserved structural integrity despite gradually progressive loss of kidney function associated with ARVD. These observations were confirmed and extended with transjugular biopsies of post-stenotic kidneys demonstrating remarkably intact tubular and glomerular structures, although glomerular volume measurements confirm reduced perfusion [32]. These investigational data provide a physiologic basis for the observation that many patients with moderate ARVD can be managed for many years with stable CKD without progressive injury [8,33].

This adaptability has limits, however. As post-stenotic flows fall to more severe levels, both kidney volume and microvascular structures are reduced. Studies using BOLD imaging confirm development of pathologic tissue hypoxia in the renal cortex and expanded medullary deoxygenation with critical loss of blood flow and renal parenchymal size [34]. When severe restrictions are in place for prolonged periods, restoration of large vessel blood flows by endovascular dilation and stent placement fails to restore microvascular structures and/or GFR in experimental studies [35]. Figure 2 illustrates this point in a swine model of ARVD that develops slowly over 6–12 weeks in a manner similar to human ARVD. These observations reinforce the failure to restore GFR consistently in human trials of revascularization [8,36]. Taken together, these data indicate that macrovascular disease and loss of parenchymal renal perfusion eventually lead to rarefaction of microvascular structures within the kidney that reduce kidney function, often irreversibly.

## 4. Tissue Hypoxia and Inflammatory Injury

Recent studies underscore the central role of inflammatory cytokines and cellular infiltration in both experimental and human renal disease. It has been suspected that ARVD may be associated with disturbed local and cellular immunity even before it affects renal blood flow [37]. Under normal conditions, almost no identifiable T cells or macrophages are visible within the kidney parenchyma. After acute injury, some degree of macrophage infiltration can be observed. Functional roles of inflammatory cells undergo complex phenotypic shifts (described as M1 to M2 variations in polarity) that modify cellular function from phagocytic to reparative structural actions, including formation of new microvessels and tubular regeneration [38,39]. The mechanisms governing T cell and macrophage functions are complex and dynamic, varying considerably under different conditions. A growing body of evidence indicates that the transition from acute kidney injury (AKI) to chronic kidney disease (CKD) reflects inflammatory pathways with phenotypic direction to fibrosis and loss of 5ubuleglomerular structural integrity [38,40].

Our studies in human subjects with ARVD underscore the association of active inflammatory injury in parallel with deepening tissue hypoxia in a post-stenotic kidney. Studies of tissue biopsies identify the infiltration of CD68+ cells in peri-glomerular and tubular structures in post-stenotic kidneys which are not present in normal kidneys (biopsies taken at implantation from normal kidney donors) [32]. When stenosis progresses to total occlusion, inflammatory infiltrates of activated T cells and macrophages are widely present throughout the ischemic kidney [41]. Sampling of renal vein blood from individual kidneys with ARVD demonstrate high circulating levels of both inflammatory [42,43] and proangiogenic cytokines [4]. Importantly, the relative levels of these markers are related quantitatively to the severity of measured tissue hypoxia and the loss of GFR in individual kidneys [4]. Such measurements reinforce the concept that as microvascular occlusion progresses to magnify tissue hypoxia within a kidney, more severe inflammatory injury pathways develop that are associated with loss of parenchymal function and viability.

## 5. Stem Cells and Injury Pathways in the Kidney

**Figure 3:**
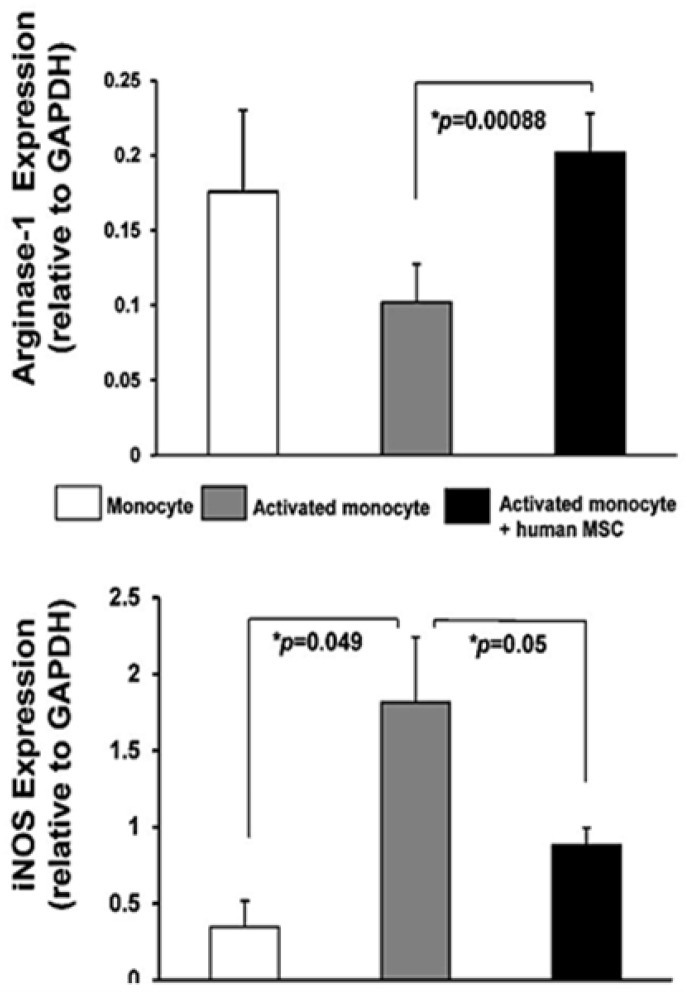
Protein expression in (white) quiescent or activated (gray, Mø) monocytes and in (black) Mø incubated with human mesenchymal stromal/stem cells (MSC). Western blotting showed increased iNOS (inducible nitric oxide synthase) (reflecting a primarily M1 phenotype) expression in activated monocytes that MSC inhibited, while arginase-1 increased (reflecting a transition to a primarily M2 phenotype). GAPDH (glyceraldehyde-3-phopsphate dehydrogenase) was used as a loading control for immunofluorescence assays. These data in vitro support the capacity for human adipose-derived MSC to modify macrophage phenotypes, consistent with the experimental data indicating such a shift is required to repair tubular integrity after AKI. Data from S. Textor and L. Lerman, with permission.

Recognition of the linkage between vascular rarefaction, inflammatory pathways and loss of kidney function raises the following question: “Can modification of these pathways abrogate the development of chronic kidney disease in ARVD?” Early studies using intrarenal administration of endothelial progenitor cells (EPC) in the swine model suggested that induced neovascularization would favor improved recovery of GFR [44]. Studies of mesenchymal stromal/stem cells (MSC) also demonstrate functional characteristics with released paracrine mediators that favor angiogenesis under ischemic conditions [45,46]. Remarkably, these MSC are also capable of modifying inflammatory cells in favor of reparative phenotypes under some conditions [40,47,48] (Figure 3). Hence, these functional properties of MSC offer the potential to favorably shape the intrarenal response to reduced blood flow in favor of both angiogenesis and reparative immunologic phenotypes. Initial studies of intrarenal administration of MSC in the swine model confirmed that these could be administered without evident toxicity and with stimulation of angiogenesis in the renal cortex well above the levels observed with restoration of vessel patency alone [49] **(**Figure 2). Recovery of renal function in experimental renovascular occlusive disease was also observed after MSC infusion both in the swine and the rodent models [50].

Our studies in human subjects were undertaken to examine this question using autologous adipose-derived MSC. We obtained an investigational new drug (IND 15082) authorization based on safety data in swine and other models to expand human cells using good manufacturing practices in the Mayo Human Cell Therapy laboratory under the direction of Dr. Allen Dietz. Cell expansion utilized proprietary human platelet-derived growth media [51], safety testing and release criteria as previously described [52]. Autologous cells were utilized to avoid possible HLA (human lymphocyte antigen) sensitization and/or cell destruction related to antigenic incompatibility. Numerous studies indicate that administered cells are widely distributed when given intravenously, with the majority being removed from circulation and a large fraction localized to pulmonary vascular sites [53]. We designed our studies to require direct intraarterial administration into post-stenotic kidneys, specifically to evaluate the effect of local delivery and to assess the potential for local toxicity related to microvascular occlusion should it occur. These studies were designed to evaluate the effects of intraarterially administered MSC both on toxicity markers in the early period after administration (weekly and monthly) and on renal hemodynamics and function when studied three months after infusion.

## 6. Functional Characteristics of Human MSC Related to Age and Hypoxia

Utilizing autologous MSC raises the issue of phenotypic and, likely, epigenetic differences between individuals based on age, gender and other properties. Studies using mRNA phenotyping indicated that both age and the underlying level of kidney function may modify MSC characteristics [54]. Hypoxia—in particular, hypoxic preconditioning—is recognized to modify multiple functional properties of these cell types. Whether the hypoxic environment of kidney tissue of ischemic nephropathy modifies the functional characteristics of MSC may affect their efficacy in vivo. We examined the changes induced by hypoxia in MSC derived both from normal kidney donors and from individuals with ARVD differing both in terms of age and the underlying level of kidney function. These data demonstrate qualitatively similar responses to hypoxia in both healthy donor and ARVD MSC with increases in growth rates and cytokine expression, although the initial levels of cytokine expression in vitro differed substantially. Studies of human adipose-derived cells obtained before and after administration of senolytic agents indicate that profiles of senescence markers can be modified considerably [54,55]. The degree to which conditioning regimens may modify in vivo activities of these cells is an important issue that remains to be fully defined.

## 7. Mesenchymal Stem Cell Effects in Human Subjects with Ischemic Nephropathy

After obtaining the Institutional Review Board approval from the Mayo Clinic College of Medicine, the initial (first-in-human) studies of patients with ARVD were undertaken to evaluate the effects of three doses of MSC (1 × 10^5^, 2.5 × 10^5^ and 5 × 10^5^ cells/kg) administered into the renal artery of a post-stenotic kidney.

**Figure 4:**
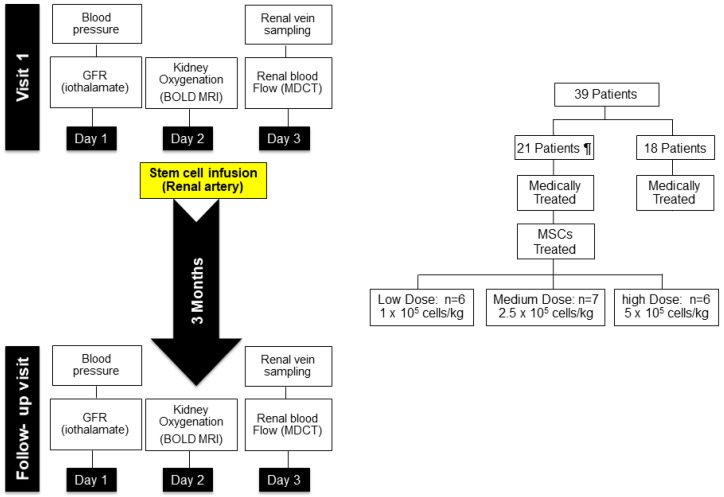
Schematic protocol (left panel) for human studies of adipose-derived autologous MSC in ischemic nephropathy. Patients were studied as inpatients during a fixed sodium intake for 3 days before undergoing renal angiography and MSC infusion into the most stenotic kidney. Studies included formal protocols for glomerular filtration rate (iothalamate clearance), tissue oxygenation (BOLD MR) and CT derived measurements of tissue volume and perfusion, in addition to renal venous sampling (see text). All of these were repeated 3 months after MSC infusion Right panel: doses of MSC infused in three cohorts as well as a control series with sequential studies under medical therapy only. Two patients (of the 21 originally) could not be treated with MSC due to occlusion of the renal artery at the time of angiography. Data from [56].

Materials and methods. Each patient was admitted to the Clinical Trials and Research Unit (CTRU) at St. Mary’s Hospital in Rochester, MN. Dietary sodium intake was maintained at 150 mEq/d daily. Day 1 included measurement of blood and urine for sodium excretion and of GFR by iothalamate clearance over three collection intervals during water hydration and determination of urinary microalbumin, total protein and markers of renal injury as previously described. Day 2 included BOLD MRI using both axial and coronal views before and after administration of furosemide (20 mg IV) as described [9,31]. Day 3 included sampling of renal vein blood during placement of the central venous catheter for determination of renal blood flow (RBF) using multidetector CT (MDCT) and renal venous sampling, which were approved by the Mayo IRB. After completion of imaging studies, autologous MSC were infused into the stenotic artery as indicated in Figure 4.

### In Vivo Imaging Studies

BOLD MRI assessed cortical and medullary oxygenation and tubular function. Acquisition of MR images with increasing echo times allows computation of their regression with the signal logarithm [20], with the slope (R2* = 1/T2*) related to deoxyhemoglobin levels and thence the blood and tissue oxygen content detected at 3T.

Image acquisition involved three transaxial slices over the two kidneys selected using scout acquisition (image series in three body axes performed for slice selection) within 15 s. A gradient multi-echo sequence acquired T2*-weighted images of the kidneys at end expiration. TR (repetition time) was 100 ms, and TE (echo time) was from 7 to 56.6 ms, with a 3.3 ms step (a total of 16 images over 15 s).

MDCT assessed bilateral, single kidney, cortical and medullary RBF, perfusion (mL/min/g) and volumes, as well as the anatomy and degree of obstruction in the stenotic renal artery.

Image acquisition involved localization scans to identify tomographic levels containing the kidneys; four mid-hilar levels were selected for flow studies. The kidneys were scanned at 20 × 1.2 collimation and 0 table-feed, a 0.5 s gantry rotation and 0.36 s partial reconstruction (B35 kernel); 45 scans (6-mm levels) were acquired 4 s after a bolus injection (0.5 cc/kg at 10 mL/s, up to 40 cc) iopamidol into the central venous catheter.

CT data analysis used the image analysis software ANALYZE™ (Biodynamic Imaging Resource, Mayo Clinic, Rochester, MN, USA) and MatLab^®^. Regions of Interest were selected from the aorta, bilateral cortex and medulla during the vascular phase, and time–attenuation curves analyzed using a mathematical model that we developed [29].

Blood samples from renal veins and inferior vena cava were collected in EDTA tubes during renal angiography. Cytokines and homing factors were measured using Millipore Luminex panels for cytokines/chemokines and angiogenic factors [56].

A specific dose for each subject was administered after protocol studies under standardized conditions summarized in Figure 4. Whether such direct administration would lead to adverse effects characterized by microvascular occlusion, tissue injury and/or systemic reaction was unknown. Changes in temperature, complete blood counts, hemoglobin, lactate dehydrogenase and sedimentation rate were followed over 12 weeks at regular intervals. After three months, these individuals returned for repeat measurements of blood pressure, individual kidney renal blood flows, and kidney oxygenation using BOLD MRI under standardized conditions as before. Importantly, sodium and medication intakes were maintained at the levels identical to those during the pretreatment study. The details of this protocol and individual measurements were published [56].

**Figure 5:**
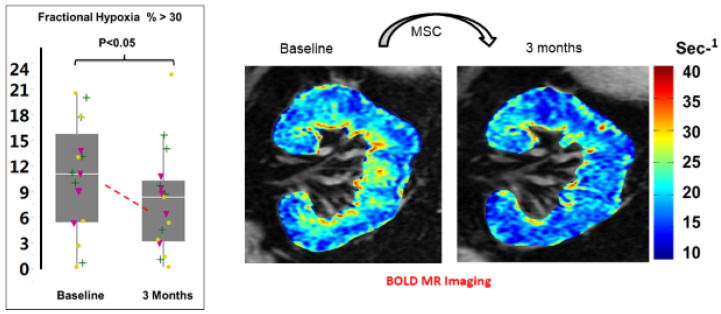
BOLD imaging of human kidneys before and three months after MSC administration (right panel) demonstrates reduction of tissue hypoxia as reflected by lower levels of deoxyhemoglobin in coronal imaging. Left panel summarizes the reduction in the fraction of whole kidney slice with R2* above 30 sec-1 in 19 patients with post-stenotic kidneys treated with autologous MSC infusion. These changes were associated with a rise in cortical perfusion and blood flow, evident both in the stenotic and non-stenotic kidneys, despite reductions in arterial pressure.

It should be emphasized that these Phase 1a studies were undertaken without endovascular dilation of the large vessel stenosis. As expected, the stenotic kidneys were characterized by reduced cortical and medullary volumes and substantially reduced renal blood flows. They were also characterized by more widespread evidence of tissue hypoxia (measured as the fraction of kidney tissue with R2* levels above 30 [28]) as compared with the contralateral kidneys. Several notable changes were seen after three months. In particular, the level of cortical perfusion (blood flow/cubic mL of tissue) rose from 2.3 (1.4–2.5) to 2.6 (1.6–3.2) ml/mi/cc tissue, *p* = 0.03. No changes in the measured volumes were observed, but the cortical and total renal blood flows rose 15–20% in the stenotic kidney due to increased perfusion (from 164 ± 99 to 190 ± 126 mL/min, *p* = 0.007). No such changes were observed in the patients undergoing the identical protocol studies without MSC administration [56,57]. Importantly, BOLD measurements after three months demonstrated reductions in the fractional hypoxia >30 s^−1^ for the stenotic kidneys (Figure 5). We interpret these findings to suggest that microvascular changes in a post-stenotic kidney allow increased cortical circulation with concomitant relief of tissue hypoxia. It is important to note that similar changes in cortical perfusion were also observed in the contralateral kidney that had not undergone MSC infusion. Cortical perfusion rose, although no changes in R2* were seen, which was already low and reflected minimal overt hypoxia in the non-stenotic kidneys. A slight rise in medullary perfusion was observed, and overall renal blood flow rose to a similar degree as that observed in the stenotic kidney. These observations suggest that systemic effects, likely from paracrine mediators and/or renal crosstalk, affected the contralateral kidney as well as the stenotic side. Again, the observed changes in the contralateral kidney were different after MSC infusion from changes in the kidneys treated with medical therapy only or endovascular stent placement [57].

All the above changes indicated that intrarenal MSC administration was associated with diminished tissue hypoxia, likely as a result of improved tissue perfusion related to microvascular repair. Whether this was the result of angiogenesis of new vessels or restoration of patency from previous collapsed vessels cannot be ascertained, although the rise in perfusion even in the contralateral kidneys with near normal blood flows would favor the former.

Blood samples from each renal vein were obtained during both the baseline and the three-month follow-up studies. For many of these kidneys, the stenotic levels of angiogenic biomarkers (angiopoietin-2, vascular endothelial growth factors A and C) were elevated as compared to non-stenotic kidneys and fell when remeasured after three months. Similarly, renal venous markers of inflammatory cytokines (neutrophil gelatinase-associated lipocalin (NGAL), interferon gamma and tissue inhibitor of metalloproteinases 2 (TIMP-2) were higher in post-stenotic kidneys and fell overall when measurements were repeated three months after MSC infusion.

## 8. Clinical Effects of MSC Administration

**Figure 6:**
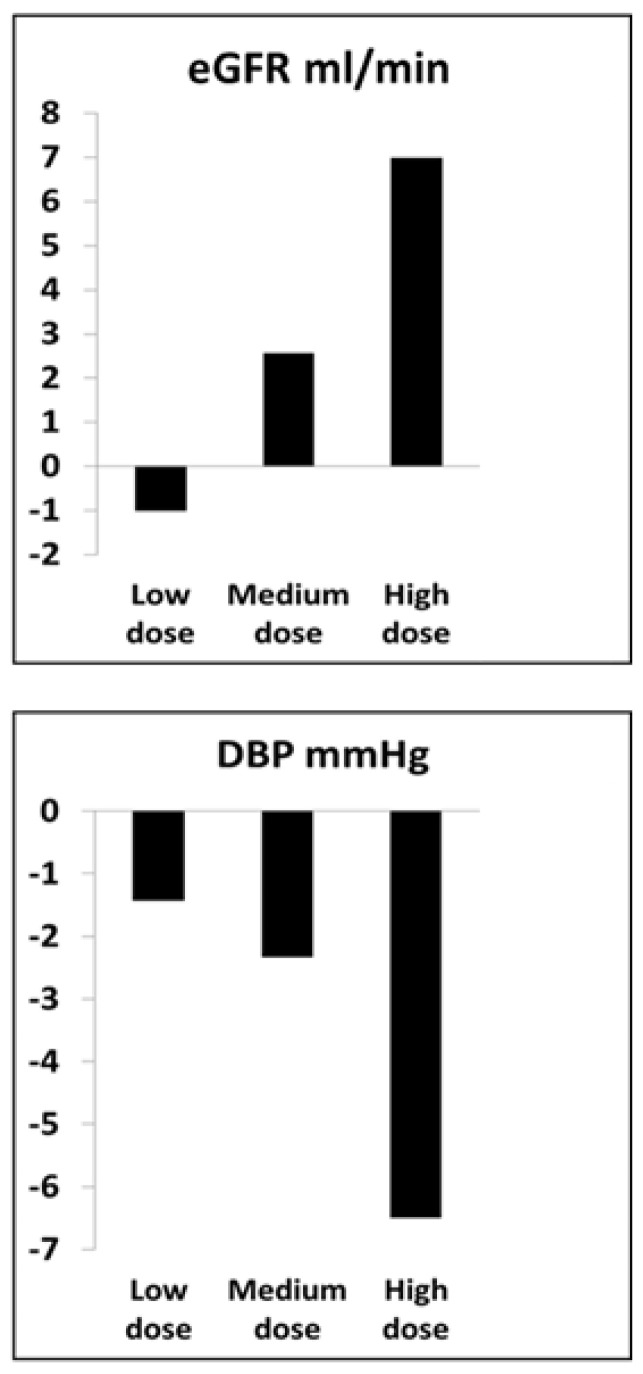
Average dose-related changes in the estimated glomerular filtration rate (eGFR) and arterial diastolic blood pressure (DBP) observed after three months in patients with ARVD treated with intraarterial infusion of autologous adipose-derived MSC. These were associated with reduced levels of tissue hypoxia and increased cortical blood flow (see text). Doses included were 1 × 10^5^, 2.5 × 10^5^ and 5 × 10^5^ cells/kg into a single renal artery.

Did MSC infusion modify kidney function and/or systemic hemodynamic effects to a meaningful degree? Measurements of temperature, complete blood counts, erythrocyte sedimentation rates and lactate dehydrogenase during the first weeks and months demonstrated no clinically evident toxicity related to arterial infusion of cell counts up to 5 × 10^5^ cells/kg, often approaching 50 million cells, directly into the kidney. Considerable care was taken to assure that antihypertensive drug therapy was unchanged during the protocol studies of this patient group. Overall systolic blood pressures fell modestly in the MSC-treated patients (144 + 11 to 136 + 13 mm Hg, *p* = 0.04), a change which mainly reflected a dose-related fall in SBP in the highest dose cohort (140 + 12 to 128 + 8 mm Hg, *p* = 0.005). Similarly, changes in measured GFR were modest, but appeared to be dose-related, insofar as the highest dose group rose from 57 + 7 to 64 + 9 mL/min, *p* = 0.045 (Figure 6). The lower dose groups had minimal changes. There was a modest reduction in urinary protein excretion, again, most evident in the highest dose cohort.

## 9. Microvascular Repair Associated with MSC Therapy in Humans

These studies provide preliminary but essential data consistent with increased microvascular perfusion associated with administration of autologous adipose-derived MSC. They extend to human subjects results from experimental studies in both swine and rodent models and support the potential role for cell-based adjunctive therapy in clinical disease. The previous studies of allogeneic MSC as a protective maneuver against acute kidney injury (AKI) were ambiguous [58,59], and a prospective trial in post-operative AKI was terminated [60]. Our studies were deliberately focused on a slowly progressing long-term microvascular disease beyond large vessel ARVD known to be prone to induce CKD. They reinforced the association of tissue hypoxia that develops with advanced disease and activation of both angiogenic and inflammatory injury of the kidney associated with progressive loss of GFR. Most importantly, they provided data that support the role of MSC as an adjunct to improve local tissue perfusion and reduce inflammatory injury. At high dose levels, our data support the potential for these changes to modestly increase glomerular filtration while reducing arterial pressure and urine protein excretion. Whether such effects can be extended to other forms of kidney injury associated with microvascular injury such as diabetes [61] and/or nephrosclerosis is an important issue that is currently under active study [62,63]. These conditions are not necessarily characterized by similar pathways of tissue hypoxia as noted with severe ARVD.

## 10. Caveats

Several important caveats deserve emphasis and further research. Some of these are summarized in Table 1. Our selection of autologous cells was based on the concern that introducing cells with different allotypes posed a risk of either allodestruction and/or allosensitization that might confuse interpretation of the results and/or pose a hazard to the patient. Some authors argue that MSC are non-immunogenic and therefore relatively protected from those concerns [63]. As a practical matter, using autologous cells is time-consuming with regard to harvest, expansion and testing for administration. These features limit their consideration for treatment related to acute processes. Perhaps most importantly, autologous cells are particularly susceptible to donor characteristics associated with age, gender, underlying disease (e.g., atherosclerotic disease, diabetes) and kidney dysfunction, all of which likely modify the potential efficacy of MSC [54]. While many properties of MSC are flexible and can be modified by the conditions under which they operate, it is likely that interindividual variability will play a large role in their actual clinical impact.

The magnitude of changes in blood flow and GFR associated with MSC infusion in these studies was modest. The clinical effect for any individual depends a great deal on the underlying degree of renal dysfunction and to what degree the entire functional kidney mass is affected. Studies of renal revascularization have identified a range of changes observed that include some improvement versus some deterioration of GFR after vascular manipulation. While these changes are complex, further deterioration of GFR for any reason is associated with a fall in long-term renal prognosis and survival [10]. Whether the minor changes in GFR, BP and/or urine protein observed in the favorable responses reported here translate into a change in prognosis remains unknown.

Further work will depend on defining the most effective dose of MSC, routes of administration and cell tracking. It is possible the repeated dosing will be required to maintain microvascular integrity, as the durability of these effects has not yet been established. Among the most important elements will be the integration of cell-based therapy with other therapeutic maneuvers. In the case of ARVD, it will be essential to evaluate the role of MSC as an adjunct to other interventions, such as renal artery revascularization, introduction of vasculogenic cytokines and/or mitochondrial protection. Some authors propose introducing angiogenic cytokines (such as vascular endothelial growth factor (VEGF) and/or hepatocyte growth factors) into the renal circulation, the microvascular effects of which increase when held in place with structural biopolymer scaffolding [64,65]. Both of the latter interventions have preliminary data to support alternative pathways for restoring microvascular structures and functional integrity under specific conditions [66,67].

## 11. Summary

Taken together, these studies demonstrate microvascular rarefaction as a primary mechanism associated with progressive injury in the human kidney associated with ARVD. They identify the limits of adaptation to reduced blood flow and a transition to overt tissue hypoxia associated with inflammatory injury. Our studies demonstrate that intrarenal infusion of autologous adipose-derived MSC is capable of increasing tissue perfusion and blood flows without removing large vessel stenosis, consistent with restoring some degree of microvascular function. These changes were associated with reduced inflammatory biomarkers in the renal veins and dose-related increments in glomerular filtration after three months. It is our view that cell-based therapy likely will be an important adjunctive resource to restore microvascular integrity in both ARVD and other renal diseases associated both with vascular rarefaction and inflammatory injury.

## Figures and Tables

**Table 1:** Caveats related to stem cell-based therapy in the kidney.

Cell preparation and conditioning Autologous vs. allogeneic cells Caveats for autologous cells:
i. Interindividual variability with varied genetic/epigenetic features ii. Age/condition-related effects iii. Optimized expansion, media, testing and verification iv. Time required for preparation/verification and administration
c Caveats for allogeneic cells:
i. Interindividual variability (as above) ii. Potential for allosensitization (considered low but possible) iii. Potential for allogeneic destruction (important to exclude if results are negative)
d Cell preconditioning to optimize efficacy, e.g., inflammatory cell polarization, homing signals, selection of subpopulations
Cell administration Localized: higher dose, transient residence, but verifiable Intravenous: likely sequestration in widespread sites, limited local dose effects Need for effective tracking over both time and location Dose relationships: initial dose:
i. Need for repeated dosing? ii. Durability of responses?
Potential development as adjunctive maneuvers
Anatomic restoration of large vessel flows Local neovascularization, e.g., angiogenic cytokine repair Associated pathways, e.g., mitochondrial protection

## Data Availability

The data presented in this study are available on request from the corresponding author.
